# Assessing the national capacity for disaster research response (DR2) within the NIEHS Environmental Health Sciences Core Centers

**DOI:** 10.1186/s12940-019-0498-y

**Published:** 2019-07-04

**Authors:** Nicole A. Errett, Erin N. Haynes, Nancy Wyland, Ali Everhart, Claire Pendergrast, Edith A. Parker

**Affiliations:** 1 Department of Environmental and Occupational Health Sciences, University of Washington School of Public Health, 1959 NE Pacific Street, Box 357234, Seattle, WA 98195 USA; 2 Department of Epidemiology, University of Kentucky College of Public Health, Lexington, KY USA; 3 Department of Occupational and Environmental Health, University of Iowa College of Public Health, Iowa City, IA USA; 4 Department of Community and Behavioral Health, University of Iowa College of Public Health, Iowa City, IA USA

**Keywords:** Disaster, Research, Environmental health, NIEHS

## Abstract

**Introduction:**

Disaster research response (DR2) is necessary to answer scientific questions about the environmental health impacts of disasters and the effectiveness of response and recovery strategies. This research explores the preparedness and capacity of National Institute of Environmental Health Sciences (NIEHS) P30 Core Centers (CCs) to conduct DR2 and engage with communities in the context of disasters.

**Methods:**

In early 2018, we conducted an online survey of CC Directors (*n* = 16, 69.5% response rate) to identify their DR2 relevant scientific assets, capabilities, and activities. Summary statistics were calculated. We also conducted in-depth, semi-structured interviews with 16 (69.5%) CC Community Engagement Core directors to identify facilitators and barriers of DR2 community engagement. Interview notes were coded and thematically analyzed.

**Results:**

*Survey:* While 56% of responding CCs reported prior participation in DR2 and preparedness to repurpose funding to support DR2, less than one third reported development of a disaster-specific data collection protocol, deployment plan, or concept of operations plan, participation in an exercise to test DR2 capacity, development of academic partnerships to conduct DR2, development of a process for fast-tracking institutional review board approvals for DR2, or maintenance of formal agreements with state, local, or community-based partner(s). A number of CCs reported developing or considering developing capacity in these areas. Barriers to, and tools and resources to enhance, CC engagement in DR2 were identified. *Interviews:* Four key components for community engaged DR2 were identified: pre-existing community relationships, responsive research that benefits communities, coordination among researchers, and coordination with community response partners. Several roles for, benefits of, and barriers to Community Engagement Rapid Response Teams (CERRT) were described.

**Conclusions:**

CCs have significant scientific assets and community partnerships that can be leveraged for DR2; however, additional planning is necessary to ensure that these scientific assets and community partnerships are leveraged when disasters strike.

**Electronic supplementary material:**

The online version of this article (10.1186/s12940-019-0498-y) contains supplementary material, which is available to authorized users.

## Introduction

Nearly all disasters have environmental health impacts [1]. Yet, events such as the Gulf Oil Spill have demonstrated that gaps remain in our understanding of the health impacts of these events and the effectiveness of interventions to minimize them [2].

With each disaster comes an opportunity to conduct high-quality research that enhances knowledge of short and long-term health impacts due to resulting environmental exposures among affected communities [3]. The rigorous and systematic collection of time-sensitive data following a disaster can enhance our understanding of disaster health impacts and the interventions used to minimize them [4]. Moreover, short-term data collected for research purposes can serve a dual purpose to inform real-time emergency response and recovery operations [4]. Despite these potential benefits, a previous lack of disaster research response (DR2) infrastructure has caused the collective environmental health sciences research community to miss several opportunities to answer critical scientific questions [2, 3]. For example, because of insufficient DR2 infrastructure, it took over 11 months to begin data collection following the Gulf Oil Spill and a year to procure funding for the study of health impacts from Hurricane Sandy [3, 4].

Specific barriers to the conduct of DR2 include: research issue identification and prioritization (e.g., community concerns); research process challenges (e.g., funding, institutional review board (IRB)); relationships, coordination, and engagement (e.g., research networks); and infrastructure and implementation challenges (e.g., training) [5]. In response, the National Institute of Environmental Health Sciences (NIEHS) and the other federal government offices and agencies (e.g., the U.S. Department of Health and Human Services’ Office of the Assistant Secretary for Preparedness and Response) have made efforts to focus on and expand disaster science infrastructure to support DR2 [3]. In particular, NIEHS has developed data collection tools and resources, hosted trainings and exercises, conducted pre-positioning planning, established funding and protocols, and addressed issues specific to rapid IRB review for time-sensitive disaster research [4, 6].

Recognizing the importance of coordination for successful DR2, NIEHS has also called for the development of disaster research networks that leverage existing research networks sponsored by NIH and other federal agencies [4]. In response, a nascent “Environmental Health Sciences Network for Disaster Response,” composed of diverse research centers, grantees, and academic partners committed to conducting DR2, has been established [1, 5].

The NIEHS Environmental Health Sciences P30 Core Centers (CC) Program funds “institutional infrastructure to support scientific equipment, facilities, and other resources that can be shared among environmental health researchers.” [7] The 23 P30 CCs (as of January 2018), strategically located across the United States [7], are well positioned to serve a leading role in the Environmental Health Sciences Network for Disaster Response. While each CC has a unique focus [7], their networked connection is supported through an annual scientific meeting, and their collaboration is promoted through NIH Administrative Supplement opportunities for multicenter projects. In addition to scientific assets, each CC has a community engagement arm that develops partnerships with communities, community-based organizations, and other stakeholder groups in their region [8].

Leveraging the significant existing scientific infrastructure, community partnerships, and networked connections of CCs to perform DR2 can synergize scientific investments. Yet, the extent to which these resources have been leveraged for DR2 preparedness has not been assessed among the 23 CCs. CCs ready, willing and able to share resources and to support one another in the event of a disaster could maximize and sustain existing NIEHS-funded research while also addressing the challenges of disaster response research.

In order to better understand what resources are available for DR2 and how to best advance and leverage them within and across the CCs, we surveyed NIEHS P30 CC Directors to identify the CC’s overall resources and capabilities that could be leveraged to support DR2. In addition, we interviewed NIEHS P30 Core Community Engagement Core (CEC) Directors, or the faculty or staff member at each CC that directs outreach and engagement efforts on behalf of the CC, to understand the challenges and opportunities of engaging community partners in DR2.

## Methods

We conducted an online survey of NIEHS P30 CCs to identify their DR2 relevant scientific assets, capabilities, and activities. The survey was administered using Qualtrics (Qualtrics, Provo, UT). An email invitation and several follow-up reminders were sent to all CC directors in January 2018, and the survey closed in mid-February. Sixteen (69.5%) CCs responded to the survey. Summary statistics were calculated using Microsoft Excel. Missing responses (i.e., item nonresponse) were excluded at the question level, and summary statistics were calculated using only the actual responses for each individual survey question. Survey questions with results reported herein are provided in Additional file 1.

We also conducted a qualitative content analysis of in-depth, semi-structured interviews with CC CEC directors or their equivalent. Key informants were selected based on their professional role; we invited the person from each CC responsible for overseeing the CC’s community engagement activities to participate by email, along with follow-up email and/or phone reminders. Sixteen (69.5%) of CEC responded and agreed to participate. At the time, two members of the research team served as the CEC Director of their CCs (EAP and EH) and abstained from participation as a key informant. Reasons for nonresponse of the other five CEC directors are unknown. As there was a finite population from which we could recruit, data saturation was not a consideration in the recruitment process. CEC directors were contacted separately and directly to participate in interviews. Twelve CCs participated in both an interview and the survey, four participated in the survey only, and four participated in an interview only, representing a total of 20 CCs.

Interviews explored facilitators and barriers of community engagement in DR2. Interviews also explored the feasibility of a Community Engagement Rapid Response Team (CERRT) that could be deployed to affected areas to support community relations necessary for DR2. Interviews lasted approximately 30 min each, and interview questions are provided in Additional file 2.

Interviews were conducted by three experienced qualitative researchers (NAE, NW, EAP). Some key informants had working relationships with interviewers related to their professional roles in environmental health research and community engagement. Interviews were recorded and detailed, point-by-point notes were taken by the interviewer and/or research assistant (AE). Recordings were referenced to provide clarity to notes on an as-needed basis, which were then used as the primary data source for the remainder of the qualitative analysis.

A preliminary codebook was developed based on the interview guide. Notes were then reviewed (NAE) and additional codes were added to include themes that emerged during interviews. The codebook was reviewed by two members of the research team that participated in interviews (NW and EAP). A single coder (NAE) applied codes to notes using N-Vivo Qualitative Research Software (QSR International Pty Ltd. Version 10, 2014). Analytic memos were developed (NAE) to synthesize key themes that emerged throughout the interviews. All members of the research team that participated in interviews (NW, EAP, AE) reviewed the analytic memos to confirm major themes were reflective, inclusive and illustrative of the interview data, as well as that minor themes and counterpoints were clearly and consistently articulated.

The University of Iowa IRB determined this study to not be human subjects research and to not require IRB approval. The University of Washington Human Subjects Division determined the survey to be not human subjects research and the interviews to be human subjects research that qualified for exempt status.

## Results

### P30 CC director survey

CCs directors reported various levels of experience with and readiness to conduct DR2 (Table 1), including participation in DR2 (56%); development of a data collection protocol (12%), concept of operations plan (19%); or deployment plan (19%); preparation to repurpose funding to support DR2 (56%); development of a process for fast-tracking IRB for DR2 (27%); participation in an exercise to test DR2 capacity (19%); development of partnerships with another Center or University to conduct DR2 (31%); and maintenance of memoranda of understanding/agreement with state, local, or community-based partner(s) (31%). A number of other CCs are developing or considering developing capacity in these areas (Table 1).

**Table 1:** P30 Core Center Capacity to Conduct DR2

Center has …^a^	Yes	No	In development or under consideration^b^
	% (n)	% (n)	% (n)
Participated in DR2	56% (9)	19% (3)	25% (4)
Developed a data collection protocol to answer disaster-specific research questions	12% (2)	50% (8)	38% (6)
Developed concept of operations plan to conduct disaster research within its state/region	19% (3)	56% (9)	25% (4)
Developed a deployment plan for researchers who go into the field outside of the CC’s geographic area to conduct disaster response research	19% (3)	69% (11)	12% (2)
Prepared to repurpose funding to support disaster response research	56% (9)	13% (2)	31% (5)
Developed a process for fast-tracking IRB of disaster response research (*n* = 15)	27% (4)	40% (6)	33% (5)
Participated in an exercise to test its capacity to conduct disaster response research	19% (3)	81% (13)	N/A
Partnered with another Center or University to conduct disaster response research	31% (5)	69% (11)	N/A
Maintained memoranda of understanding/agreement with a state, local, or community-based partner(s)	31% (5)	50% (8)	19 (3)

^a^Missing responses (i.e., item nonresponse) were excluded at the question level, and summary statistics were calculated using only the actual responses for each individual survey question. For any question where less than all 16 participating CCs responded, the total number of item-level responses is provided in the first column

^b^N/A indicates that “In development or under consideration” was not a response option

Several barriers to conducting DR2 were reported (Table 2). At least one quarter of the responding CCs reported each of the following items as moderate or severe barriers to DR2 participation: funding (*n* = 10), faculty capacity (*n* = 6), training (*n* = 6), staff capacity (*n* = 5), pre-developed protocols (*n* = 5), planning (*n* = 4), lack of experience (*n* = 4), and IRB (*n* = 4).

**Table 2:** P30 Core Center Reported Barriers to Conducting DR2

	No barrier (1)	Minor barrier (2)	Neutral (3)	Moderate barrier (4)	Severe barrier (5)	Mean
	% (n)	% (n)	% (n)	% (n)	% (n)	
Expertise	50% (8)	19% (3)	13% (2)	19% (3)	0	2
Equipment	38% (6)	31% (5)	31% (5)	0	0	1.9
Laboratories	50% (8)	19% (3)	19% (3)	6% (1)	6% (1)	2
Staff capacity	13% (2)	25% (4)	31% (5)	25% (4)	6% (1)	2.9
Faculty capacity	19% (3)	25% (4)	19% (3)	31% (5)	6% (1)	2.8
Training	13% (2)	13% (2)	38% (6)	38% (6)	0	3
Planning	13% (2)	25% (4)	38% (6)	19% (3)	6% (1)	2.8
Pre-developed protocols	13% (2)	25% (4)	31% (5)	25% (4)	6% (1)	2.9
IRB	25% (4)	13% (2)	38% (6)	25% (4)	0	2.6
Experience	13% (2)	44% (7)	19% (3)	25% (4)	0	2.6
Geographic location	38% (6)	6% (1)	44% (7)	13% (2)	0	2.3
Funding	0	13% (2)	25% (4)	19% (3)	44% (7)	3.9

The need for tools and resources to enhance the capacity of CCs to conduct DR2 was also identified (Table 3). At least half of CCs reported that funding for each of the following items would have a major impact on their ability to conduct DR2: funding for program development (*n* = 9) and funding to support faculty development (*n* = 8). At least half of responding CCs reported the following training types would have a major or moderate impact on the ability of to conduct DR2: disaster research design (*n* = 8), disaster research implementation (*n* = 8), health and safety while conducting disaster research (*n* = 8), mental and behavioral health during DR2 (*n* = 9), and the Incident Command System (*n* = 9). In addition, at least half of responding CCs reported each of the following supports as having moderate or major impacts on their ability to conduct DR2 (Table 3): availability of exercise support (*n* = 9), planning templates (*n* = 9), funding to purchase equipment (*n* = 10), funding to support laboratories (*n* = 9), funding to support staff development (*n* = 14), and tools and resources to develop disaster-resource partnerships (*n* = 10).

**Table 3:** Tools or resources that impact a Core Center’s ability to conduct DR2^a^

	No impact (1)	Minor impact (2)	Neutral (3)	Moderate impact (4)	Major impact (5)	Mean
	% (n)	% (n)	% (n)	% (n)	% (n)	
IRB support	25% (4)	6% (1)	31% (5)	25% (4)	13% (2)	2.9
Training on disaster research design	6% (1)	31% (5)	13% (2)	44% (7)	6% (1)	3.1
Training on disaster research implementation	0	25% (4)	25% (4)	38% (6)	13% (2)	3.4
Training on health and safety while conducting disaster research	6% (1)	31% (5)	13% (2)	38% (6)	13% (2)	3.2
Training on mental and behavioral health during disaster response research (*n* = 15)	0	20% (3)	20% (3)	33% (5)	27% (4)	3.7
Training on the Incident Command System	0	13% (2)	31% (5)	38% (6)	19% (3)	3.6
GIS capabilities	25% (4)	25% (4)	38% (6)	13% (2)	0	2.4
Exercise support (*n* = 15)	0	13% (2)	27% (4)	47% (7)	13% (2)	3.6
Planning templates (*n* = 15)	0	27% (4)	13% (2)	53% (8)	7% (1)	3.4
Funding for program development	0	0	6% (1)	38% (6)	56% (9)	4.5
Funding to purchase equipment	0	0	38% (6)	25% (4)	38% (6)	4
Funding to support laboratories (*n* = 14)	0	0	36% (5)	21% (3)	43% (6)	4.1
Funding to support faculty development (*n* = 15)	0	0	7% (1)	40% (6)	53% (8)	4.5
Funding to support staff development	0	6% (1)	6% (1)	44% (7)	44% (7)	4.3
Tools and resources to develop disaster research partnerships	0	6% (1)	31% (5)	44% (7)	19% (3)	3.8

^a^Missing responses (i.e., item nonresponse) were excluded at the question level, and summary statistics were calculated using only the actual responses for each individual survey question. For any question where less than all 16 participating CCs responded, the total number of item-level responses is provided in the first column

### Interviews with CEC Directors

CEC Directors identified the following themes relevant to DR2 work. CEC Directors are interested in working on a variety of hazards and DR2 topics. Key informants expressed interest in understanding the public’s experience and concerns following a disaster, as well as in identifying and leveraging existing data sources, identifying data needs, and initiating more robust data collection when there is no available data. Key informants described topical interests in long-term health impacts and exposures; inequities of impacts and impacts of cumulative exposures; the impact of disasters on public housing residents; and the political economy and political ecology of disaster relief efforts.

Most CEC Directors do not have in-depth knowledge of the NIEHS DR2 initiative, but several expressed that they, their CC, or researchers currently affiliated with their CC conducted disaster research in a variety of contexts, including wildfires, hurricanes, flooding events, terrorist events, water contamination events, oil spills, and chemical spills. Research and community outreach efforts were focused on a variety of content areas, including air quality, plume modeling, healthcare access, worker health, and children’s health. Research and community engagement activities during these events included exposure assessment, sampling, surveying, needs assessment, and risk communication. CECs with prior engagement in DR2 emphasized their role in risk communication and information translation.

CEC Directors are interested in additional DR2 training. While those not interested in or involved with DR2 would likely not be interested in DR2 training, CEC Directors in general supported the development of additional training and felt that it would be useful to themselves or others at their CC. Suggested training included: overview of the DR2 process; working with communities and tribes; Incident Command System; occupational health; coordination across multiple research groups; communicating DR2; psychological resilience; legal issues in DR2; DR2 study design; and institutional review board issues in DR2.

Successful DR2 rests on the backbone of strong, pre-existing community relationships. Key informants consistently described the value of existing relationships in DR2 implementation. When communities view researchers as a resource, information can be rapidly translated and communicated in the context of a disaster response. These relationships can also facilitate the identification of post-event community priorities, and identification of research questions that are relevant to community concerns. Further, grant reviewers like to see that relationships are established.

Since trusted researcher-community relationships take a long time to develop and disaster research requires a rapid response, it is important that these relationships are developed before a disaster strikes. By having trusted relationships in place, it was noted that there would be less of a “learning curve” for both the researchers and the community. One key informant recommended that DR2 community engagement plans be developed in advance of a disaster.

While deep community knowledge and personal trusted relationships were described as highly valuable, one key informant noted that communities that work with researchers regularly may be more receptive to working with other researchers because of their familiarity and experience with the research process. Researchers may also “transfer” their trusted relationships to other researchers; for example, a community may welcome a researcher that is introduced or accompanied by a researcher with whom they have a pre-existing relationship. However, researchers may be inclined to protect the relationships that they have spent time developing. Competition among CCs further disincentivizes transferring trusted relationships.

The value of CECs in connecting scientists with communities was described and has been demonstrated in prior events. However, it was noted that the CEC role is to address community needs and connect communities with information, not to conduct research. The use of CEC networks to connect communities with researchers should be approached with caution to avoid negatively impacting trusted relationships built over many years.

Disaster researchers should coordinate with existing community preparedness and response structures, and ensure research goals, processes, and limitations are transparent and beneficial to impacted communities. Communities that have been impacted by a disaster have many immediate and competing needs, and the short and intermediate-term benefits of DR2 might not be immediately apparent to affected communities. The goals of the research, research processes (e.g., IRB), and their limitations should be clearly and intentionally communicated to the community.

Key informants emphasized that disaster researchers are not autonomous, and should integrate local response structures and/or incident command systems into their response plan(s). Information communicated to the public should also be coordinated with these entities. In the absence of this type of coordination, miscommunication or misaligned priorities may ensue. One key informant described a potential conflict between disaster response and disaster research. Another described the need for conflict resolution between researchers, research institutions (related to IRB), and between researchers and communities.

Researchers should get to know local response organizations prior to a disaster, and DR2 plans should be made in advance with community involvement. It was noted that communication systems are likely to be disrupted in a disaster, and back-up communication systems must be planned. One key informant described working closely with his/her institutional preparedness officer as a way to help understand whole community engagement in disaster management. Another key informant identified a tabletop exercise as an opportunity to integrate with community response organizations.

Key informants described the value of engaging with specific community organizations about DR2. Examples include: riding along with the fire department to get buy-in around collaboration to collect data following a disaster; engaging local health departments; and working with the clinical enterprise.

Financial, administrative, and logistical issues may impact successful DR2. Funding immediate research needs was described by several CC Directors as a barrier to DR2. One key informant noted that NIEHS time sensitive R21 grants [9] function the way that they are intended, and that all details do not need to be known prior to submissions; however, another noted that the timeline of these awards poses challenges. Delays in commencing DR2 can reduce its relevance. Researchers are hesitant to collect samples if they do not know whether they will have the money to analyze them.

The need for funding for disaster-related CEC work was also noted. Although the NIEHS Partners in Environmental Public Health (PEPH) network was described as helpful in connecting disaster-impacted communities with previously developed resources, CECs are largely left to use their existing resources to meet additional disaster-specific community engagement needs.

Advance planning with IRBs was described as an essential component of effective DR2. Developing “blanket IRBs” and understanding IRB transference issues in advance of a disaster were identified as opportunities to streamline DR2.

The value of having a compendium of resources for DR2, including willing personnel, available instruments, and existing expertise in some specific areas, was described. One key informant desired a “go bag” of tools (e.g., survey instruments) ready to go in advance of a disaster. One key informant mentioned making sure that people going into a disaster know that they might not come back healthy or alive, and should make appropriate arrangements (e.g., create a will, identify a healthcare proxy, share account information).

Coordination among disaster researchers, as well as competitive relationships among researchers and CCs, can be impediments to DR2. One key informant suggested that a cooperative process to research, rather than use of competitive grants, could foster coordination. For example, NIEHS could convene a group of researchers and facilitate a consensus-building discussion about what should be done in response to a disaster.

**Fig. 1:**
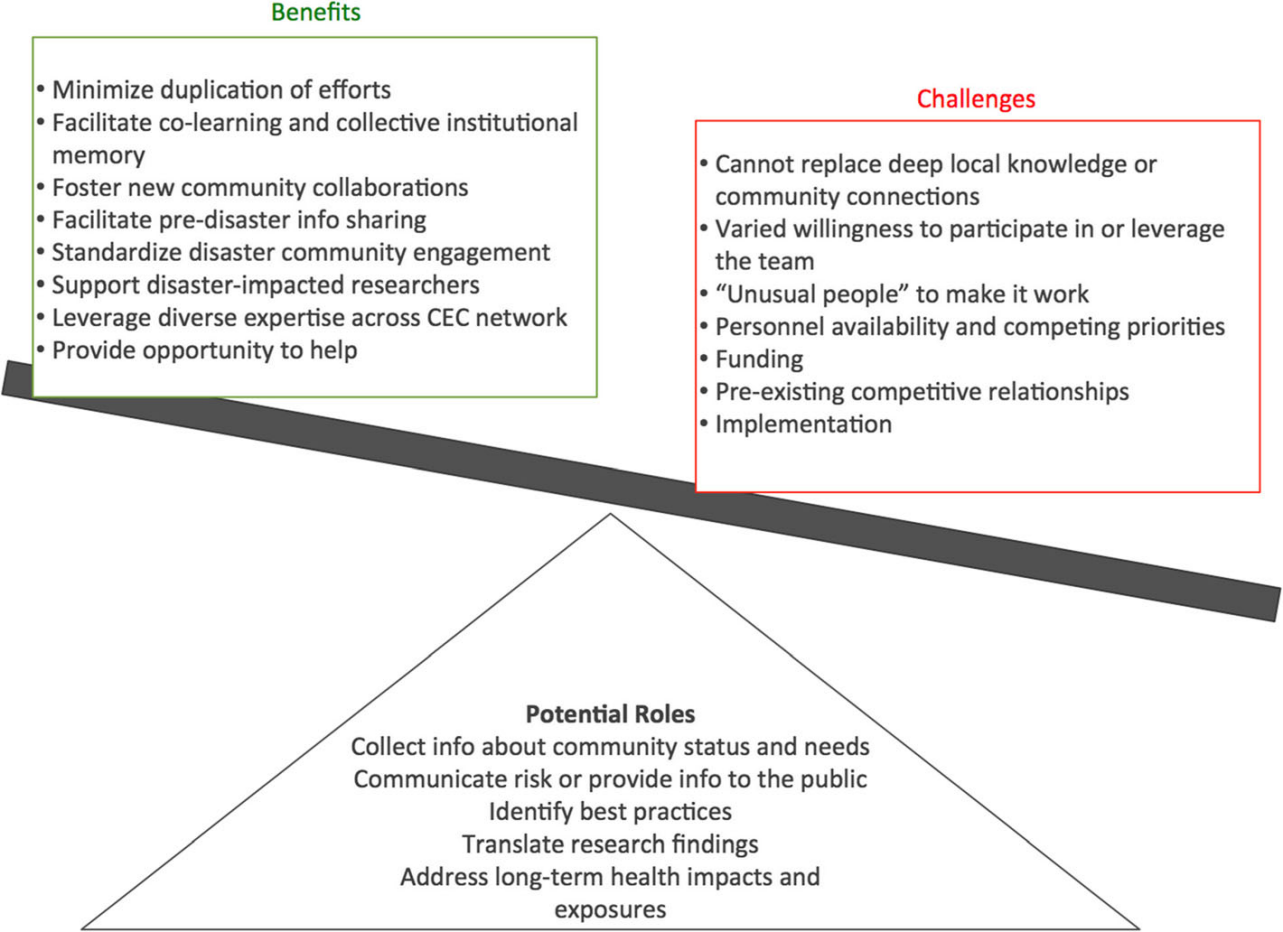
Potential Roles, Benefits and Challenges of Community Engagement Rapid Response Teams (CERRTs)

Formation of Community Engagement Rapid Response Teams (CERRT) is a potentially valuable DR2 resource worthy of further exploration (Fig. 1). CERRTs could serve a variety of functions in the context of a disaster response, including collecting information about community status and needs, messaging, best practice identification, communicating interim research findings, and identifying and responding to health impacts of long-term exposures. CERRTs could help to minimize duplication of efforts, facilitate co-learning, improve collective institutional memory about disasters, and foster new community collaborations. CERRT could inform the development of standardized community engagement tools or approaches, facilitate information sharing among CECs before a disaster, and host forums at specific national meetings (e.g., at the Society for Advancement of Chicanos/Hispanics and Native Americans in Science’s annual meeting) about engagement of particular community groups.

CERRTs were also identified as a resource for researchers affected by a disaster. In such circumstances, local researchers could continue to serve as intermediaries between communities and CERRTs, but could have help while they deal with personal impacts of the disaster.

It was emphasized that CERRTs could not replace relationships between CECs and communities. CERRTs, or other external parties, should be deployed at the invitation of the community and accompanied by the researcher/CEC with pre-existing community connections. CERRT response should be part of a sustained community engagement strategy.

Several potential issues around CERRT implementation were reported, and require additional exploration. CERRTs were identified as a mechanism to leverage expertise and experience across the CEC network, and to provide an opportunity for people who want to help but don’t know how. Yet, there is a need to identify individuals willing to be part of a team. CEC Directors expressed varied willingness to serve as a member of a CEC team and to leverage the team in the context of a response in which they were the “lead” (i.e., local CEC with relevant community connections). One key informant noted that it would take unusual people to make it work. Expertise in rapid response was identified as a need. Most CEC personnel have multiple responsibilities outside of the CEC, and may not have time to devote to a CERRT effort. Furthermore, individual availability at the time of an event could be a barrier to participation. Competing funding and scientific priorities, including existing funded projects, could deter involvement.

Resources and materials necessary to implement CERRTs must be identified. For example, would participation be in a volunteer capacity? Competition among CCs was described as a barrier to CERRTs. Administrative Supplements to existing NIH grants were described as an opportunity to overcome this barrier and promote coordination for this purpose.

Implementation of CERRTs at a regional level was also discussed. For example, regional or local individuals could be hired to work on community engagement, or CECs in close geographical proximity could work together in the field and consult remotely with CECs located farther away.

## Discussion

Our survey indicates that although NIEHS P30 CCs have a variety of scientific assets, experience, and expertise that can be mobilized for DR2, few have developed or tested plans to successfully participate in DR2. However, nearly a quarter of the 16 surveyed CCs have plans in development, indicating that CC DR2 capacity will increase.

While significant barriers to CC engagement in DR2 were identified, opportunities to enhance capacity were highlighted, including training, funding, and guidance. The NIEHS DR2 program has made significant progress developing and making available DR2 tools, training and guidance [6]. Moreover, since 2010, NIEHS has offered a time-sensitive funding mechanism to provide resources for conducting DR2 in the aftermath a disaster [9]. However, funding opportunities to develop CC capacity can be enhanced, including through Administrative Supplements to existing NIH grants. NIEHS may also consider funding an NIEHS DR2 coordinating center, and/or encouraging “DR2 cores” as part of CC’s optional other facility cores [10]. Moreover, integrating faculty with DR2 experience or expertise into CC operations may increase sustained DR2 capabilities, and the overall DR2 capacity of the environmental health sciences research network..

Disasters may also create unique challenges for CCs located in affected areas, including impacts to research facilities and availability of personnel. For example, students and faculty were not allowed to return to the University of Puerto Rico (UPR) in Mayagüez for 40 days following Hurricane Maria [11]. In response, a variety of universities stepped up to help the UPR faculty and students continue their research and education [11]. Similarly, by promoting the continuity of science operations, collaborative relationships have the potential to protect NIEHS investments and the national environmental health enterprise. Yet, only 31% of CCs responding to our survey indicated they had established partnerships with other universities for DR2 (Table 1). In order to protect, employ and synergize these investments in the context of disaster, CCs should develop collaborative relationships and agreements to support one another and other NIEHS-funded research.

**Fig. 2:**
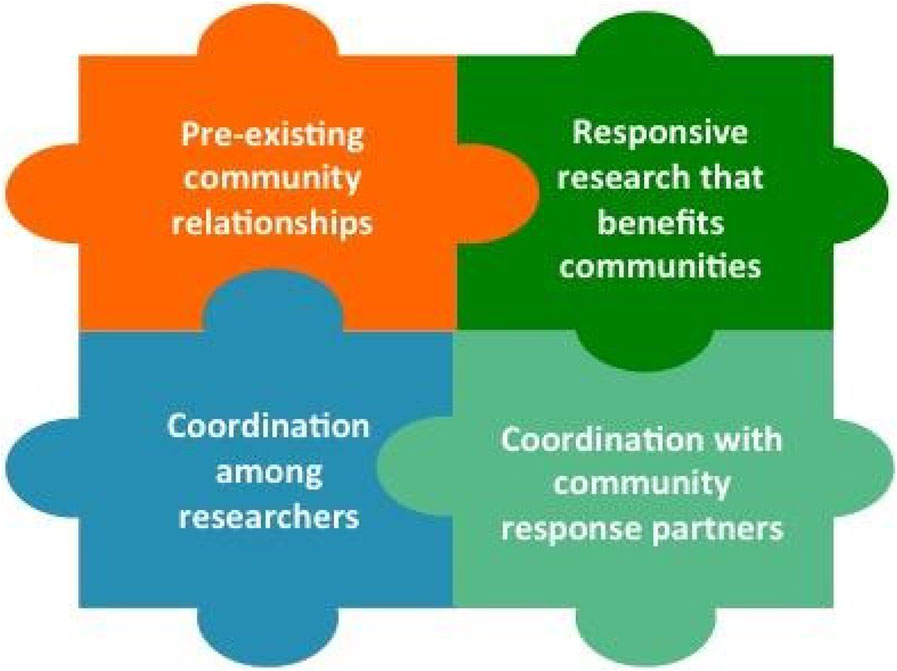
Core Components of Community Engaged DR2

Our interviews indicate that pre-existing community relationships, responsive research that benefits communities, coordination among researchers, and coordination with community response partners are core components of community-engaged DR2 (Fig. 2). Advanced planning can enhance environmental health related disaster research and provide immediate, direct benefit to affected communities through technical assistance and outreach. CC CECs should work with local, state, and tribal partners to plan for their role in disaster response.

The possibility of CERRTs as a specific strategy to promote cross-CC support and collaboration was explored during key informant interviews. CEC Directors were enthusiastic about the development of CERRTs and identified numerous opportunities to use them to promote effective community engagement in the context of disaster. However, they concurrently identified numerous considerations and potential challenges related to resourcing and implementation (Fig. 1). We recommend the structure and operationalization of CERRTs be further explored through a workshop or working group. Given CC CECs’ experience and expertise in engaging communities on environmental health science issues, CEC leadership should be involved in any planning activities to determine how to best leverage the CCs in CERRTs; for example, by training and rostering CEC staff to serve on CERRTs.

During interviews, key informants suggested specific steps that researchers should take prior to deploying to a disaster area, including developing a “go bag” of research tools and developing a will. The NIEHS Worker Training Program developed the Researcher Deployment Guide to support researchers and academic/research organizations before, during and after deployment [12]. The guide describes steps that researchers and academic organizations can take to prepare themselves and their families pre-deployment, includes helpful checklists, and explains what researchers can expect in a disaster environment, including information about how disaster responses are organized and managed. We recommend that researchers and academic organizations review such guidance prior to DR2 deployments.

### Limitations

Our study focused exclusively on NIEHS P30 CCs, and findings may not be generalizable to the entire environmental health research enterprise. Moreover, our catalogue of resources, readiness and interest does not account for the vast resources available through, and potential contributions of, other NIEHS-funded centers (e.g., Superfund Centers or Children’s Environmental Health Centers), individual NIEHS-funded research projects, or research supported by tribal, state or local governments, other NIH institutes or federal entities (e.g., CDC or NSF), or private funding (e.g., foundations). We did not receive responses from all CCs, and CCs that did not respond may be systematically different than those that did. In addition, we did not provide interview participants with interview data or analysis products for review and comment (i.e., “member-checking”), which could have enhanced the trustworthiness of our qualitative content analysis. Finally, this cross-sectional assessment does not capture the continual evolution of CC assets, interests, and readiness for DR2.

## Conclusions

NIEHS P30 CCs have significant scientific assets and community partnerships that can be leveraged to conduct research in the context of a disaster response in order to enhance our understanding of the health impacts of disasters and to identify effective strategies to mitigate them. However, additional planning and coordination is needed to ensure that those CCs that are willing and able to bring these assets to bear in the face of the next disaster are also ready to do so. Additional technical assistance and resources can provide low-cost opportunities to advance the capacity of CCs to participate and collaborate in DR2.

## Additional files

Additional file 1: Core Center Director Survey Questions for Reported Data. (DOCX 20 kb)

Additional file 2: Community Engagement Core Director Interview Questions. (DOCX 15 kb)

## Data Availability

The data collected and used during the current study may be made available from the corresponding author on reasonable request.

## Abbreviations

CC: Core Center
CEC: Community Engagement Core
CERRT: Community Engagement Rapid Response Team
DR2: Disaster Research Response
IRB: Institutional Review Board
NIEHS: National Institute of Environmental Health Sciences
