# Supplementary material for: Assessing the national capacity for disaster research response (DR2) within the NIEHS Environmental Health Sciences Core Centers
Source: Environ Health. 2019 Jul 4;18:61. doi: 10.1186/s12940-019-0498-y (PMC6610905; doi:10.1186/s12940-019-0498-y)
Supplement: Supplementary file 1 — Core Center Director Survey Questions for Reported Data. (DOCX 20 kb) [file 12940_2019_498_MOESM1_ESM.docx]

**Additional file 1: Core Center Director Survey Questions for Reported Data**

Has your Center previously participated in disaster response research?

[Yes/No/No, but we’re thinking about it]

Does your Center have a pre-developed data collection protocol that can be used to answer disaster-specific research questions?

[Yes/No/Under development]

Does your Center have a concept of operations plan to conduct disaster research **within your state/region**?

[Yes/No/Under development]

Does your Center have a deployment plan for researchers that go into the field **outside of your geographic area** to conduct disaster response research?

[Yes/No/Under development]

Is your center prepared to repurpose funding to support disaster response research?

[Yes

No

Unsure]

Does your institution have a process for fast-tracking IRB of disaster response research?

[Yes/No/In progress]

Which of the following exercises has your Center participated in to test your capacity to conduct response research?*

[Center-level exercise

University-level exercise

Community-level exercise
We have not participated in an exercise]

*option to check all that apply

Does your Center have partnerships with another Center or University to conduct disaster response research?

[Yes/No]

Does your Center maintain memorandums of understanding/agreement with any of your state, local, or community-based partners?

[Yes/No/Under Development]

Which of the following are barriers to your Center's ability to conduct disaster response research?

|  | 1-No barrier | 2- Minor barrier | 3- Neutral | 4 – Moderate barrier | 5- Severe barrier |
| --- | --- | --- | --- | --- | --- |
| Expertise |  |  |  |  |  |
| Equipment |  |  |  |  |  |
| Laboratories |  |  |  |  |  |
| Staff capacity |  |  |  |  |  |
| Faculty capacity |  |  |  |  |  |
| Training |  |  |  |  |  |
| Planning |  |  |  |  |  |
| Pre-developed protocol |  |  |  |  |  |
| IRB |  |  |  |  |  |
| Experience |  |  |  |  |  |
| Geographic location |  |  |  |  |  |
| Funding |  |  |  |  |  |
| Other (please specify) ____________________ |  |  |  |  |  |
| Other (please specify) ____________________ |  |  |  |  |  |

Which of the following tools/resources would impact your Center’s ability to conduct disaster response research?

|  | 1-No barrier | 2- Minor barrier | 3- Neutral | 4 – Moderate barrier | 5- Severe barrier |
| --- | --- | --- | --- | --- | --- |
| IRB support |  |  |  |  |  |
| Training on disaster research design |  |  |  |  |  |
| Training on disaster research implementation |  |  |  |  |  |
| Training on health and safety while conducting disaster research |  |  |  |  |  |
| Training on mental and behavioral health during disaster response research |  |  |  |  |  |
| Training on the incident command system |  |  |  |  |  |
| GIS capabilities |  |  |  |  |  |
| Exercise support |  |  |  |  |  |
| Planning templates |  |  |  |  |  |
| Funding for program development |  |  |  |  |  |
| Funding to purchase equipment |  |  |  |  |  |
| Funding to support laboratories |  |  |  |  |  |
| Funding to support faculty development |  |  |  |  |  |
| Funding to support staff development |  |  |  |  |  |
| Tools and resources to develop disaster-research partnerships |  |  |  |  |  |
| Other (please specify) ____________________ |  |  |  |  |  |
| Other (please specify) ____________________ |  |  |  |  |  |
