# Supplementary material for: Assessing the national capacity for disaster research response (DR2) within the NIEHS Environmental Health Sciences Core Centers
Source: Environ Health. 2019 Jul 4;18:61. doi: 10.1186/s12940-019-0498-y (PMC6610905; doi:10.1186/s12940-019-0498-y)
Supplement: Supplementary file 2 — Community Engagement Core Director Interview Questions. (DOCX 15 kb) [file 12940_2019_498_MOESM2_ESM.docx]

**Additional file 2: Community Engagement Core Director Interview Questions**

1. Can you tell me about your role with the CEC?
   1. How long have you worked with your CEC? Do you have any other duties with the Center?
2. Can you tell me about your CEC? Can you describe the communities with which you work and where they are located?
3. Can you describe how familiar you are with the NIEHS DR2 research initiative?
4. What kinds of disasters do you see, or expect to see, in your region?
5. Has your center been involved in DR2 research in the past? (IF NO, SKIP TO QUESTION 6)
   - - If so, can you describe the research?
     - How was your CEC involved in the research? How was your community involved in the research?
     - What are the lessons learned you would like to share with other CECs and Centers about DR2 research?
6. What do you see as barriers to community engagement in DR2 research? How about facilitators to community engagement in DR2 research?
7. One idea we are exploring with these interviews is the idea of tapping into CEC staff across the country to develop “rapid response teams” that could be deployed to affected areas to support community outreach and education around environmental hazards of the disasters.
   - - What are your initial thoughts about this idea? Barriers? Facilitators?
8. As part of this project, we will conducting a training on DR2 research. Would you be interested in this training? Are there areas that you feel we should cover in this training? Do you have suggestions of presenters for this training?
